# Targeting the Microenvironment in High Grade Serous Ovarian Cancer

**DOI:** 10.3390/cancers10080266

**Published:** 2018-08-10

**Authors:** Nkechiyere G. Nwani, Livia E. Sima, Wilberto Nieves-Neira, Daniela Matei

**Affiliations:** 1 Department of Obstetrics and Gynecology, Northwestern University, Chicago, IL 60611, USA; Nnwani@northwestern.edu (N.G.N.); Livia.sima@northwestern.edu (L.E.S.); wilberto.nieves-neira@nm.org (W.N.-N.); 2 Robert H. Lurie Comprehensive Cancer Center, Chicago, IL 60611, USA

**Keywords:** high-grade serous ovarian cancer, tumor microenvironment, angiogenesis, immune response, metastasis, therapeutic targeting strategies

## Abstract

Cancer–stroma interactions play a key role in cancer progression and response to standard chemotherapy. Here, we provide a summary of the mechanisms by which the major cellular components of the ovarian cancer (OC) tumor microenvironment (TME) including cancer-associated fibroblasts (CAFs), myeloid, immune, endothelial, and mesothelial cells potentiate cancer progression. High-grade serous ovarian cancer (HGSOC) is characterized by a pro-inflammatory and angiogenic signature. This profile is correlated with clinical outcomes and can be a target for therapy. Accumulation of malignant ascites in the peritoneal cavity allows for secreted factors to fuel paracrine and autocrine circuits that augment cancer cell proliferation and invasiveness. Adhesion of cancer cells to the mesothelial matrix promotes peritoneal tumor dissemination and represents another attractive target to prevent metastasis. The immunosuppressed tumor milieu of HGSOC is permissive for tumor growth and can be modulated therapeutically. Results of emerging preclinical and clinical trials testing TME-modulating therapeutics for the treatment of OC are highlighted.

## 1. Introduction

**Figure 1:**
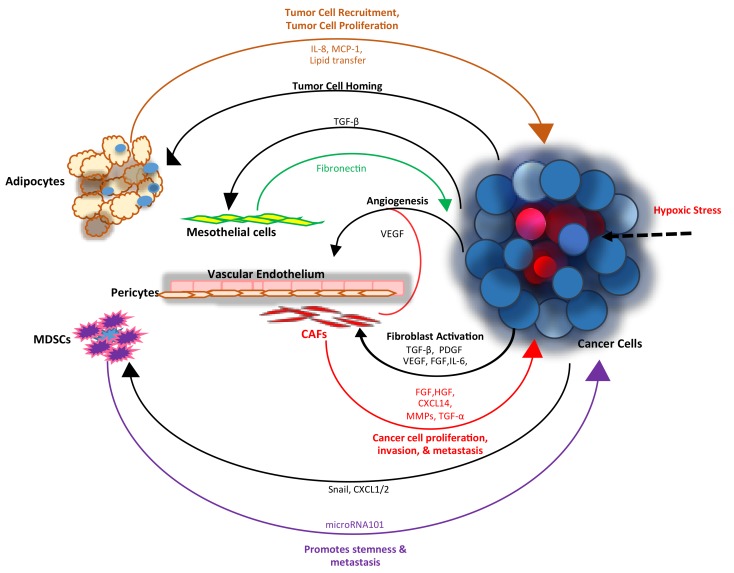
The interplay between cancer and stromal cells in the tumor microenvironent TME regulates tumor growth and metastasis: as tumors grow, hypoxic stress and low nutrient availability drives the release of tumor-secreted growth factors and cytokines that exert paracrine effects on the surrounding stroma. Sustained exposure to tumor-derived transforming growth factor-β (TGF-β), platelet-derived growth factor (PDGF), fibroblast growth factor (FGF) and vascular endothelial growth factor (VEGF) drives fibroblasts trans-differentiation into (cancer associated fibroblasts) CAFs. These factors also act upon endothelial cells, pericytes and immune cells to stimulate angiogenesis. CAF-derived FGF and hepatocyte growth factor (HGF) promote tumor cell proliferation, CAF-derived matrix metalloproteinases (MMPs) promote invasion while chemokine ligand 14 (CXCL14) and transforming growth factor-α (TGF-α) enhance metastasis. Ovarian cancer (OC) cell-derived TGF-β1 upregulates fibronectin secretion in mesothelial cells, which in turn enhances spheroid adhesion to the peritoneal wall. Adipocytes facilitate cells proliferation by providing energy dense lipids to the metastasized cancer cells. Cancer cells expressing Snail and chemokine (C-X-C motif) ligand 1/2 (CXCL1/2) recruit myeloid-derived suppressor cells (MDSCs) to the tumor site; conversely MDSC-secreted microRNA101 reprograms tumor cells to a stemness phenotype.

High-grade serous ovarian cancer (HGSOC) comprises the majority of epithelial ovarian tumors, is associated with a p53-mutated signature and is characterized by initial sensitivity to platinum and a unique pattern of dissemination in the peritoneal space. The peritoneum consists of mesothelial cells that cover and protect the viscera. The sub-peritoneal stroma contains a collagen-based matrix, activated fibroblasts, blood vessels, and lymphatics. This unique milieu permits accumulation of factors secreted by both cancer and stromal cells and enables metastatic seeding and tumor proliferation. The immune component of the peritoneal milieu consists of monocytes/macrophages and cytotoxic T cells. Several studies have demonstrated an “activated” phenotype of the peritoneal environment associated with ovarian cancer (OC), as opposed to its quiescent state in benign conditions [1]. The pro-inflammatory signature associated with cancer favors angiogenesis and exerts chemotactic and protective effects on cancer cells. Chemokines, cytokines, and growth factors commonly secreted in the tumor microenvironment (TME) include the stromal cell-derived factor (SDF1), interleukin-6 (IL-6), interleukin (IL-8), monocyte chemoattractant protein 1 (MCP1), Chemokine (C-C motif) ligand 5 and 7 (CCL5 and CCL7), transforming growth factor-β1 TGF β1, tumor necrosis factor-α (TNFα), fibroblast growth factor (FGF), and others [1,2,3,4]. While tumor cells play a role in the secretion of factors that modulate angiogenesis, non-transformed tumor infiltrating cells such as fibroblasts, myeloid cells, immune cells, and endothelial precursors also play a crucial role modulating neo-vascularization [5]. OC metastasis commonly involves the omentum, an adipocyte-rich organ. Lipid transfer between adipocytes and cancer cells mediated by fatty acid binding protein 4 (FABP4), through a “symbiotic” process between cancer cells and the fatty microenvironment was described as a key regulator of peritoneal metastasis [6]. As the rich TME protects cancer cells from noxious stimuli promoting tumor growth (Figure 1), its disruption through targeted therapy could arrest cancer progression. Indeed, over the past decade, several classes of novel agents targeting the ovarian TME have been developed and tested clinically. The most active agents are antiangiogenic therapies, which have been recently approved by the Food and Drug Administraton FDA for OC. Other emerging strategies, particularly immunotherapy, are in various stages of development. Here, several targeted therapies directed against the main components of the TME will be reviewed.

## 2. Fibroblasts

Fibroblasts represent the preeminent cellular component of connective tissues, the structural scaffold of many organs in the body. They are a heterogeneous population of mesenchymal-derived cells that maintain the composition of the extracellular matrix (ECM) [7,8]. As such, fibroblasts produce and deposit most of the proteins that comprise the ECM, including collagens, proteoglycans, tenascin, fibronectin, and laminin. Tissue homeostasis involves a tightly orchestrated balance of ECM synthesis and metabolism; in addition to ECM production, fibroblasts are also responsible for matrix metabolism. They produce several ECM-degrading matrix metalloproteinases (MMPs) and their inhibitors, tissue inhibitors of metalloproteinases (TIMPs) [9]. It has been observed that fibroblasts within the tumor milieu are phenotypically similar to activated fibroblasts associated with granulating tissue (wound healing) [10]. These cancer-associated fibroblasts (CAFs) function as tumor-promoting cells; playing important roles in tumor initiation and progression [11,12,13]. Although resident fibroblasts are a major source of CAFs, they can also arise from the trans-differentiation of other cell populations including epithelial cells, endothelial cells, pericytes, adipocytes and bone marrow-derived mesenchymal stem cells [14]. During tumorigenesis the trans-differentiation of the aforementioned cells into CAFs is driven by sustained exposure to tumor-derived factors including TGF-β, PDGF-BB, basic fibroblast growth factor (bFGF), vascular endothelial growth factor (VEGF), as well as microRNAs, reactive oxygen species (ROS), matrix metalloproteases (MMPs) and extracellular vesicles [15,16,17,18,19]. 

Current evidence suggests the mechanisms/downstream effectors that coordinate CAF activation vary and are contingent on CAF origin. For example, it was shown that SKOV3 cells stimulate normal fibroblasts conversion through TGF-β mediated induction of ROS and CLIC4, which led to the subsequent increase in the expression CAF markers αSMA and FAP. On the other hand, Jeon et al., demonstrated that cancer cell-derived lysophosphatidic acid induced TGF-β in adipose tissue-derived mesenchymal stem cells which then promoted their trans-differentiation into CAFs [18,20]. Likewise, expression of HOXA9, a differentiation related gene, was linked to paracrine secretion of TGF-β2 by OC cells, inducing adipose and mesenchymal stem cells to become CAFs [21]. It is unknown whether other stromal cells such as pericytes and endothelial could also contribute to the reactive stroma associated with HGSOC. 

The role of fibroblasts in cancer progression is complex. Early studies provided evidence that fibroblasts possess anti-tumorigenic function by forming a restrictive stroma. However, the atypical cancer-stroma interactions promote fibroblasts to develop tumor-permissive properties [22,23,24]. Recent reports illustrate how the reciprocal cancer cell–fibroblast communication potentiates tumor growth and progression in OC models. For example, CAFs have been shown to suppress the immune response through miR141/200a-mediated expression of CAF-derived CXCL12. This chemokine promotes infiltration of immunosuppressive CD25^+^ FOXP3^+^ T lymphocytes in the HGSOC milieu, which in turn allows tumor growth [25]. CAFs have also been shown to drive tumor cell proliferation, migration and invasion by producing high amounts of mitogenic factors, hepatocyte growth factor (HGF) and FGF [26,27,28]. Additionally, CAF-secreted IL-8 and SDF-1 drive angiogenesis to facilitate oxygen and nutrients delivery to the tumor tissue [29,30]. Fibroblasts treated with SKOV3-derived extracellular vesicles acquired an activated phenotype; in turn these fibroblast enhanced tumor and endothelial cells proliferation [17]. In another study, OC cell-derived TNF-α induced TGF-α transcription in stromal fibroblasts. In turn, TGF-α secreted by these fibroblasts promoted metastasis via induction of EGFR signaling in cancer cells [31]. CAFs also produce metabolites that are essential to cancer cells’ survival, such as lactate that is absorbed and utilized by oxidative phosphorylation in adjacent cancer cells [32]. The chemokine ligand 14 (CXCL14) is a CAFs secreted protein that is associated with a poor prognosis in OC. It was discovered that CXCL14 induced LINC00092 expression in OC cells, which resulted enhanced metastasis. LINC0009 interacted with 6-phosphofructo-2-kinase/fructose-2,6-biphosphatase 2 (PFKFB2) to induce a glycolytic phenotype in ovarian cancer cells. These interactions are necessary for maintaining the CAF-phenotype, thereby unearthing a positive feedback loop between CAF-cancer cells interactions that sustain a tumor-permissive microenvironment [33].

Cancer invasion and metastasis is also closely associated with MMPs secreted by CAFs and tumor cells and increased MMP expression has been associated with poor prognosis for various cancers [34]. In addition to modifying the ECM, MMPs can facilitate tumor growth and invasion by increasing the bioavailability of ECM tethered growth factors. For instance, CAF-secreted matrix metalloproteinase-13 (MMP-13) enhanced tumor cells invasion through proteolytic cleavage of matrix-bound VEGF and angiogenesis [35]. 

An additional factor involved in CAF-tumor cell cross-talk is the fibroblast activation protein (FAP). FAP is exclusively expressed on activated fibroblasts, and increased expression is associated with poor prognosis in many tumors [36]. In OC, FAP promoted HO-8910PM tumor cell proliferation, invasion and migration via interactions with integrin α3β1 and urokinase-type plasminogen activator receptor (uPAR) signaling complex [37]. Moreover, elevated stromal FAP expression was a strong predictive marker of platinum resistance and relapse [38]. Due to the adverse effects of CAFs on cancer recurrence and patient survival, there has been extensive investment in developing strategies to effectively target CAFs. 

## 3. Therapies Targeting Fibroblasts

FAP is overexpressed in many epithelial cancers including OC, and its expression is often associated with poor prognosis [36,38], cancer cell migration, invasion and immunosuppression [39,40,41]. As such, FAP has emerged as a potential therapeutic target to abate the tumor promoting effects of CAFs. The catalytic activity of FAP was shown to be necessary for tumor proliferation. However, inhibition of FAP enzymatic activity by small molecules has had little success in clinical trials [42,43]. In a transgenic mouse model, targeted depletion of FAP-expressing CAFs resulted in increased cancer cell death. Mechanistically, this effect was dependent on TNF-α and IFN-γ, which are known to be involved in CD8^+^ T cell mediated cancer cell death [41]. Furthermore, pre-clinical studies using vaccines against FAP showed promising results for colon and lung cancer. Vaccines targeting FAP-expressing cells significantly suppressed tumor growth by eliciting CD8^+^ or a combined CD8^+^ and CD4^+^-T cell response respectively [40,44].

TGF-β, a cytokine abundantly secreted by fibroblasts and detectable in ascites fluid, contributes to the development of a tumor-promoting microenvironment. Several TGF-β targeting agents have been evaluated in clinical trials. These include small molecule kinase, antisense oligonucleotides, and TGF-β-ligand traps [45,46]. In a mouse model of peritoneal metastasis, the TGF-β inhibitor A-83-01 improved overall survival [47,48]. Likewise, the transforming growth factor-β receptor 1 (TβRI) kinase inhibitor galunisertib inhibited tumor growth in a partly TME-dependent manner in various PDX tumors [49]. TGF-β inhibitors have also been shown to enhance the efficacy of conventional therapeutics. For example, combination treatment with TGF-β receptor inhibitor LY2109761 and cisplatin significantly blocked the growth of cisplatin-resistant ovarian xenografts [50]. Despite promising initial preclinical results, advancement of TGF-β signaling inhibitors to the clinical arena has been slow, marred by initial concerns over systemic (cardiac) toxicity, which fortunately appears to be limited in humans [51].

Several other tyrosine kinase inhibitors (TKI) have been employed to mitigate the pro-tumorigenic effects of growth factors secreted by fibroblasts in the tumor milieu, such as the platelet derived growth factor (PDGF) and fibroblast growth factor (FGF). PDGF-D over-expression was associated with lymph node metastasis and platinum resistance in ovarian cancer [52] and imatinib, a PDGFR inhibitor, was shown to inhibit OC cell growth [53]. While the precise effects of imatinib on ovarian stroma are not well defined, previous research demonstrated that this TKI suppressed angiogenesis in cervical tumors [54]. Dasatinib, another FDA approved TKI, which also targets the PDGF receptor has been shown to partially revert lung cancer-derived CAFs to a normal phenotype [55]. Clinical trials tested the PDGFR inhibitors imatinib and sorafenib in patients with recurrent platinum resistant OC and demonstrated modest clinical activity [56,57]. 

## 4. Angiogenesis

Angiogenesis is the process whereby new blood vessels sprout from the pre-existing vasculature. Angiogenesis is a tightly regulated and transient process observed in biological processes such as development, wound healing and reproduction [58]. However, pathological angiogenesis is a rate-limiting event in metastasis. As tumors increase in size (>1–2 mm^2^), nutrient and oxygen availability are reduced and an angiogenic switch is activated; the newly formed blood vessels are able to deliver nutrients and oxygen necessary for cancer cell proliferation, facilitate waste expulsion, and also provide the primary route by which cancer cells migrate to secondary sites (metastasis) [59]. In fact, tumor vascularity serves as an indicator of metastatic potential for many cancers with highly vascularized tumors having greater incidence of metastasis and reduced survival [60,61]. In cancers, angiogenesis is driven by reduced levels of anti-angiogenic factors, and sustained overproduction of pro-angiogenic molecules by tumor and host cells [58]. Angiogenesis is triggered by growth factors such as VEGF, PDGF, (FGF), angiopoietin (Ang), as well as the chemokines IL-8 and interleukin-6 (IL-6) [59,62,63]. The association between HGSOC and an angiogenic signature was recognized more than two decades ago and has remained a staple in the study of this tumor’s biology. VEGF is the most extensively studied angiogenic factor in pathological angiogenesis; it is overexpressed in HGSOC and secreted into malignant ascites [64,65,66,67]. Increased VEGF expression is associated with reduced survival rates in patients with OC [68,69,70]. In a cohort of 222 HGSOC specimens, high levels of VEGF-A were correlated with increased microvessel density and with infiltration by immune cells [71]. Interestingly, high levels of VEGF-A were associated with BRCA-mutated ovarian tumors [71]. Although cancer cells are a major source of angiogenic factors, non-neoplastic cells (immune cells, adipocytes, and CAFs) in the TME also produce the angiogenic factors required to sustain tumor growth and progression [72]. As such, there has been considerable focus on developing therapeutics to inhibit the angiogenic signaling as a means of mitigating cancer progression. 

## 5. Anti-Angiogenic Therapy (AAT)

VEGF is the most extensively studied pro-angiogenic factor and therapies targeting this pathway use either inhibition of the ligand or of its receptor, vascular endothelial growth factor receptor (VEGFR). VEGF-A is a secreted glycoprotein that belongs a family of related growth factors that includes VEGF-B, VEGF-C, VEGF-D and VEGF-E and placental growth factor (PLGF), which have varying functions in angiogenesis [73]. The VEGF system functions as a mitogenic factor for endothelial cells, induces endothelial cell migration and differentiation, and protects immature endothelial cell against apoptosis [74,75]. VEGF exerts these functions by binding to the tyrosine kinase receptors VEGFR-1 (Flt-1) and VEGFR-2 (KDR/Flk-1) on the cell surface, causing them to dimerize and become activated [76]. Bevacizumab (Avastin, Roche, Basel, Switzerland), is a humanized monoclonal antibody against VEGF that binds and inactivates VEGF, thus inhibiting endothelial cell activation and proliferation. Bevacizumab was shown to reduce tumor growth and prolong survival in murine ovarian cancer models [77,78]. Clinical trials using bevacizumab as a single agent and in combination with other therapeutics have been successful and bevacizumab is currently FDA approved for use in the front-line setting, as well as in recurrent disease [79,80].

The first clinical trial to test the efficacy of bevacizumab in OC was performed by the Gynecologic Oncology Group (protocol GOG 170D) and tested the drug in 62 patients with recurrent, platinum-resistant disease. In this trial, 21% of patients exhibited objective clinical responses and 40.3% survived progression-free for at least 6 months. Median progression-free survival (PFS) and overall survival (OS) were 4.7 and 17 months respectively [81]. This initial success led to the development of combination therapies using bevacizumab with chemotherapy. In the ICON7 phase III trial, the efficacy of bevacizumab in combination with platinum and paclitaxel was tested in patients with advanced or metastatic epithelial ovarian cancer after cytoreductive surgery. Bevacizumab was continued for 12 additional cycles or until progression of disease. Progression-free survival at 42 months was increased from 22.4 months with chemotherapy alone to 24.1 months with combination treatment (*p* = 0.04). Interestingly, PFS and OS were most significantly increased in patients at high risk for progression. In this group, survival at 42 months was 28.8 months for patients receiving standard therapy vs. 36.6 months for patients receiving carboplatin/platinum and bevacizumab [82]. Similar results were observed in GOG protocol 218, where chemotherapy plus bevacizumab followed by bevacizumab maintenance improved PFS (but not OS) compared to platinum and paclitaxel alone after cytoreductive surgery [79]. In another randomized phase III clinical trial (AURELIA Trial), bevacizumab in combination with physician’s choice chemotherapy was tested in women with recurrent platinum-resistant OC. The median PFS was 3.4 months for patients who received chemotherapy alone versus 6.7 months for patients treated with bevacizumab and chemotherapy [83]. These results summarized in Table 1 led to the approval and widespread clinical use of the first therapy targeting the ovarian cancer TME.

Other modalities to block this pathway are in development. For example, aflibercept is a recombinant fusion protein of VEGFR1 and VEGFR 2 extracellular domain, which functions as a decoy receptor and inhibits VEGF-mediated signaling by trapping VEGF-A, VEGF-B, placental growth factor-1 (PlGF-1) and (PlGF-2). Aflibercept was shown to reduce ascites and decrease the peritoneal dissemination of OC xenograft models [53,84,85,86]. A phase II trial tested the efficacy of aflibercept in patients with advanced platinum-resistant OC and malignant ascites. Patients who required three or more previous paracenteses per month were given intravenous aflibercept 4 mg/kg every two weeks. The primary study endpoint was repeat paracentesis response rate (RPRR), and a response was defined as a minimum two-fold increase in time to repeat paracentesis compared with the baseline interval. Ten out of 16 patients treated achieved a response; RPRR was 62.5% (95% CI 35.4–84.8%). Median time to repeat paracentesis was 76.0 days (95% CI 64.0–178.0), 4.5 times longer than the baseline (16.8 days) and the median PFS was 59.5 days (95% CI 41.0–83.0) [87], demonstrating that targeting this growth factor in the TME leads to appreciable clinical benefits.

However, angiogenesis is a complex phenomenon tightly regulated by complementary and cross-talking pathways, which allows for the development of resistance [88]. Thus, inhibitors that concurrently block multiple receptors were tested in an effort to improve the efficacy of AAT. Cediranib (AZD2171, AstraZeneca) is a receptor tyrosine kinase inhibitor that inhibits vascular endothelial receptor 1–3 (VEGFR 1–3), platelet-derived growth factor-α and β (PDGFR-α and -β), and c-kit. A phase II clinical trial assessed the efficacy of cediranib in patients with recurrent gynecologic cancers who had received less than two lines of platinum-based chemotherapy. Of 46 patients treated, eight patients (17%) had partial responses (PR), six patients (13%) stable disease (SD), and there were no complete responses (CRs) [89]. In another phase II trial, the efficacy of single-agent cediranib was assessed in 74 patients with persistent/recurrent OC following one round of platinum-based chemotherapy. The patients were stratified into two groups; 39 platinum-sensitive (PL-S) and 35 platinum-resistant (PL-R), and the primary endpoint was objective response rate at 16 weeks. In the platinum sensitive (PL-S) group, 10 patients (26%) demonstrated partial responses (PR) and 20 (51%) had stable disease (SD). There were no confirmed PR in the platinum resistant (PL-R) group and 23 patients (66%) had SD. The median PFS was 7.2 months for PL-S and 3.7 months for PL-R groups, and the median OS was 27.7 and 11.9 months respectively [90]. Currently cediranib is being evaluated in combination with olaparib, a poly (ADP-ribose) polymerase PARP inhibitor in women with recurrent OC. 

Nintedanib is another tyrosine kinase inhibitor for VEGFR-1-3, FGFR 1-3, PDGFR α and β. Nintedanib was tested as maintenance treatment after chemotherapy in a randomized trial. PFS at 36-weeks was 5.0% vs. 16.3% in placebo and nintedanib treated patients [91]. However, in a subsequent phase III trial (AGO-OVAR 12) nintedanib combined with platinum-based therapy did not induce a significant survival advantage after debulking surgery. The median PFS was 17.2 vs. 16.6 months for patients treated with nintedanib and placebo, respectively. A post-hoc analysis showed that nintedanib and platinum-based therapy combination improved PFS in non-high-risk patients [92]. Pazopanib (GW786034) is tyrosine kinase inhibitor for VEGFR-1, -2 and -3 PDGFR-α and -β and c-kit. An ongoing clinical phase II trial (MITO-11) is evaluating the safety and activity of pazopanib in combination with paclitaxel in patients with platinum-resistant or refractory OC. The median progression-free survival was 3.5 months in patients treated with weekly paclitaxel vs. 6.3 months in patients treated with weekly paclitaxel and pazopanib. The median overall survival was 14.8 months in paclitaxel treated vs. 18.7 months in patients treated with paclitaxel and pazopanib [93]. In all, these and other trials have convincingly demonstrated the activity of AAT in HGSOC, leading to the approval of bevacizumab for treatment in both the adjuvant and recurrent settings. New trials are evaluating the efficacy of anti-angiogenic drugs in combination with immune modulators or PARP inhibitors for treatment of gynecologic malignancies. 

## 6. Interactions with the Mesothelial Matrix

**Figure 2:**
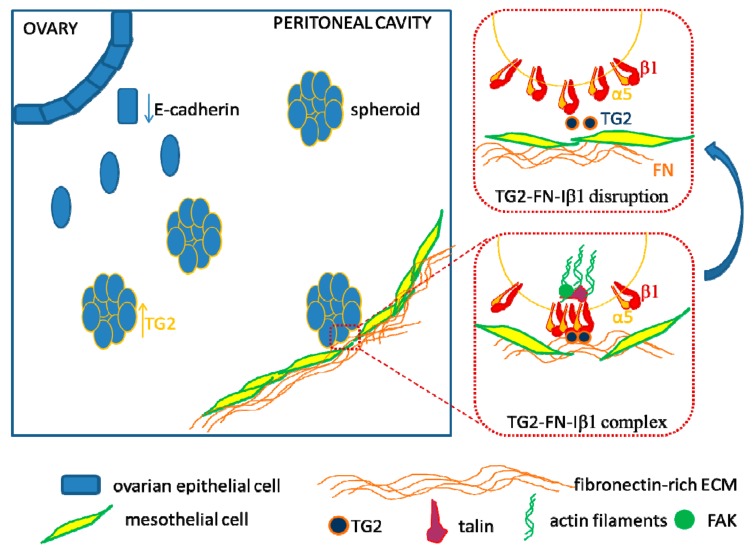
Ovarian cancer cells adhere to the mesothelial lining during tumor dissemination in the peritoneal cavity. Upon activation of EMT (epithelial-to-mesenchymal transition), cells progressively shed from the primary tumor into the peritoneal cavity (blue square). During the EMT process, there is a decrease in E-cadherin expression and increase in proteins associated to a mesenchymal phenotype, such as vimentin, tissue transglutaminase (TG2) and integrins. Cells that survive in the environment of the peritoneal cavity form spheroids. Spheroids attach to the fibronectin (FN) rich matrix secreted by the mesothelial cells, clear the subjacent monolayer and invade the underling tissue. These adhesion and invasion processes are mediated by interactions of integrin-β1 receptors with the FN fibrils in the ECM. Upon FN binding, α5β1 integrin receptors undergo clustering, which is enhanced by molecular bridges with TG2. Next, talin is recruited to the adhesion complex and provides the necessary traction force for the mesothelial monolayer displacement (red dotted bottom square). Also, “outside-in” signaling downstream of β1 integrin is activated, inducing focal adhesion kinase (FAK) phosphorylation. Therapeutic strategies targeting the TG2-FN-Iβ1 complex aim at interfering with the cell adhesion process and consequently preventing OC metastasis (red dotted top square).

In order to form secondary tumors, disseminated OC cell spheroids floating in the peritoneal cavity rely on their capacity to adhere to the mesothelial lining covering the peritoneal cavity and abdominal organs. During dissemination from the primary site, OC cells lose E-cadherin expression (Figure 2, upper left) and upregulate α5 integrin, which was proposed as a therapeutic target [94]. Secondary site invasion occurs upon displacement of the mesothelial monolayer cells (Figure 2, lower right), with cancer cells invading and submerging into the subjacent environment. The clearance of mesothelial cells is enabled by traction forces mediated by myosin and generated by the adhesion complex molecules, α5 integrin and talin-1, and is more efficiently accomplished by reprogrammed mesenchymal-like OC cells [95,96]. Other receptors that play a role in OC cell adhesion to mesothelium include CD44 and β1 integrin (Iβ1) [97]. OC cell-derived TGF-β1 upregulates fibronectin (FN) expression in mesotheial cells [98]. The adhesion of OC cells to the FN matrix secreted by mesothelial cells [98] is dependent upon α5β1 integrin clustering and talin recruitment to stabilize the adhesions (Figure 2) [95]. Integrin clustering is induced by secreted tissue transglutaminase (TG2), which forms a bridge connecting Iβ1 and FN together at the cell surface [99]. This event induces downstream RhoA activation and suppression of Src–p190RhoGAP signaling. A focus of our laboratory’s work was to understand the role played by the TG2-Iβ1-FN ternary complex in the process of OC metastasis and to test it as a new therapeutic target. By using OC orthotopic and ip xenografts, we showed that TG2 knock-down blocked peritoneal dissemination of ovarian tumors through a mechanism dependent on β1-integrin mediated cell adhesion and signaling [100,101]. Our recent results also demonstrate that engagement of integrin β1 facilitated by TG2 activates β-catenin signaling and stemness associated pathways in in vivo and organoid models of HGSOC [102,103]. 

## 7. Targeting Ovarian Cancer Cell Adhesion to the Peritoneal Matrix

Several strategies have been tested in an effort to block OC peritoneal dissemination. Treatment with blocking antibodies against integrins and the CD44 receptor were shown to inhibit OC cells adhesion to the mesothelial layer for short time intervals [104,105,106]. As α5β1 integrin is expressed on both OC cells as well as on the endothelial cells forming microvessels [107], it was expected that targeting this heterodimer (Figure 2, top square) will interfere with tumor growth and metastasis in many types of solid cancers, including OC [108]. Currently several drugs targeting integrins are under development (reviewed in [109]).

Volociximab, a chimeric antibody that binds α5β1 integrin with high affinity, was shown to block growth and dissemination of OC xenograft models [94]. However, the phase II clinical trial testing volociximab in patients with recurrent, platinum-resistant OC failed to demonstrate benefit although the drug was well tolerated [110]. Intetumumab (CNTO-95), a human αv-integrin specific monoclonal antibody that targets both αvβ3- and αvβ5-integrins showed anti-tumor and anti-angiogenic effects in xenografts models of breast cancer [111,112]. In a phase I clinical trial including patients with advanced solid tumors, one patient with ovarian carcinosarcoma had stable disease for six months [113]. Other integrin-blocking antibodies, such as etaracizumab, the humanized version of anti-αvβ3-integrin LM609 had minimal therapeutic benefit in other cancers [114]. Cilengitide is a stable cyclic pentapeptide containing an Arg-Gly-Asp (RGD) motif which allows selective binding to αvβ3 and αvβ5 integrins [115]. Cilengitide was tested in brain tumors and was found to not increase OS in glioblastoma patients during a phase III trial [116]. Given that αvβ3 integrin expression by tumor cells correlates with a favorable prognosis in OC patients [117], targeting this integrin might be a less appropriate strategy for OC. The initial disappointment with integrin targeting strategies may be related to their prior testing in the recurrent, advanced setting as single agents. Development of combination regimens and testing of these blocking antibodies in patients with low volume metastatic disease might overcome the lack of clinical success with this intervention.

FN is one of the most abundant ECM proteins in the omentum and peritoneum [118]. Adhesion of OC cells to FN via α5β1 integrin impacts “outside-in signaling” by inducing phosphorylation of focal adhesion kinase (FAK) either directly [119] or through c-Met [108]. This can further lead to activation of mitogenic pathways [120] which support tumor growth [121]. The β1 integrin–FN interaction is further enhanced by the bridging activity of TG2, a protein we discovered to be overexpressed in OC [122]. Previous work in our group has emphasized the importance of TG2 in the OC metastatic process, by providing evidence of its involvement in promoting OC cells’ epithelial-to-mesenchymal transition through activation of non-canonical NF-κB [123], increasing cell proliferation by regulating β-catenin signaling [102], enhancing peritoneal dissemination [100], and increasing invasion by regulating MMP-2 [124]. As proof of principle that the TG2-FN-Iβ1 complex represents an interesting target in OC, we used a function-blocking antibody which targeted the FN binding domain of TG2, and showed that this antibody blocked OC spheroid proliferation and tumor initiating capacity by disrupting the interaction between OC stem cells and their niche [103]. 

To discover potent and selective TG2-FN inhibitors we used both virtual docking and high throughput screening strategies. Through an initial in silico docking approach, we identified a small molecule inhibitor capable of disrupting this complex and of blocking cancer cell adhesion to the FN matrix [125]. Subsequent efforts used an AlphaLISA-based assay adapted to high-throughput screening and applied to the ChemDiv library leading to the discovery and validation of several small molecules [126]. One hit selected from this screen (TG53) was validated in vitro to be an efficient inhibitor of OC cell adhesion to FN, migration and invasion. Future efforts focus on optimizing this compound through structure–activity relationship-based strategies to generate more selective, potent and drug-like compounds which block the TG2-FN protein–protein interaction and ultimately prevent OC metastasis. 

## 8. Tumor Immune Response in Ovarian Cancer 

Preclinical models and retrospective cohort analyses of human tumor specimens have demonstrated that the interaction between cancer cells and the host immune defense plays an important role harnessing tumor progression. There are several immune cell subsets relevant for tumor progression and response to immunotherapy [127]. These are classified in two categories: immune reactive and immune suppressive cells. The immune reactive cells include primarily cytotoxic T lymphocytes and activated CD4^+^ T cells. The immune suppressive cells are myeloid lineage subpopulations known as myeloid-derived suppressor cells (MDSCs), tumor-associated macrophages (TAMs, especially M2 subtype), dendritic cells (DCs) and the lymphocyte subsets of T helper cells (Th2 subtype) and T regulatory cells (Tregs). A seminal study showed that the presence of CD3^+^ tumor infiltrating lymphocytes (TILs) in OC is associated with increased survival [128]. The 5-year overall survival (OS) was 38% for patients whose tumors contained T cells compared to 4.5% for those whose tumors were devoid of T cells. Subsequently, a strong association between the presence of CD8^+^ TILs and favorable clinical outcomes of HGSOC was recognized [129,130,131]. The CD8^+^ to T regulatory (Tregs) cells ratio was also shown to correlate with increased survival of OC patients [130]. More recently, the presence of CD8^+^ cells expressing the TNFR-family receptor CD137 (4-1BB) was reported as a prognostic marker associated with improved survival of OC patients [132]. A recent study evaluated the immune TME landscape in differentially growing metastases after several therapy cycles in an OC patient and reported heterogeneity in immune infiltrates that explained the evolution of tumor masses over nine years period [133]. This unique report revealed a correlation between the regressing or stable metastases and the presence of oligoclonal expanding T cells. Conversely, progressing tumors showed a lack of infiltration with anti-cancer lymphocytes. This study reinforces the importance of the tumor immune microenvironment to the outcome of OC disease. In all, these and other studies [134] strongly support the role of anti-tumor immunity as a key regulator in the evolution of the disease. 

Enhancing the naturally occurring immune defense could therefore play an important role harnessing disease progression. Immunotherapy has demonstrated efficacy in various malignancies [135,136]. Several immune modulatory approaches (vaccines, IL2, CTLA-4 directed antibodies, adoptive transfer of activated T cells) have been tested in OC, with promising results in early interventions [137,138]. However, the impact of immunotherapy on the survival of OC patients remains unproven and predictive markers of positive outcomes remain undefined, highlighting the need to further optimize such strategies. 

## 9. Immune Checkpoint Inhibitors

Recent advances have brought attention to the programmed cell death protein-1 (PD-1) mechanism used by cancer cells to evade immune surveillance, which can be effectively targeted by inhibitory antibodies [139]. This strategy demonstrated impressive clinical activity in several solid tumors (melanoma, lymphoma, renal, lung, and bladder cancer) leading to new FDA-approved interventions [140,141,142]. PD-1 signaling blocks T-cell activation keeping nascent T-cells in check and preventing immune responses against normal tissues. During cancer progression, this inhibitory pathway is activated by upregulating the expression of PD ligands (PD-L1 and PD-L2) on tumor and immune cells and permits evasion from immune surveillance [139]. The significance of the PD1 pathway to OC progression has been investigated; however, the emerging evidence is conflicting. On one hand, initial studies showed that the increased PD-L1 expression in ovarian tumors correlates with decreased intra-tumoral CD8^+^ lymphocytes and worse patient survival [143]. Presence of dendritic cells expressing PD1 in the OC microenvironment was also found to be associated with decreased numbers of TILs and suppressed T cell activity [144], consistent with the concept that PD-L1 represents an escape mechanism. On the other hand, more recent studies using specific PD1 and PD-L1 detection antibodies provide evidence to the contrary. Two reports showed that expression of PD-L1 on immune cells in the tumor milieu, including on tumor associated macrophages (TAMs), is associated with increased total numbers of TILs and better survival in HGSOC [145,146]. It remains unresolved how expression of the PD1 pathway elements can be causally linked to a favorable prognosis in OC. It is possible that expression of PD-L1 reflects an active immune TME (defined by increased TILs density) able to attack and eliminate the tumor, or that PD-L1^+^ TILs have a yet to be defined regulatory role in the immune response mechanism. Additional support for clinical interventions targeting this pathway includes that PD-1/PD-L1 blockade restored anti-tumor immunity in an OC xenograft model [147]. Two recent clinical trials tested PD-1 (pembrolizumab) and PD-L1 (avelumab) inhibitory antibodies in women with recurrent OC, reporting response rates of 11% (pembrolizumab) and 10% (avelumab), with 23% and 40% additional patients experiencing stable disease, respectively [148,149]. These early data suggest that immune checkpoint blockade in OC has defined, albeit modest activity. 

Another emerging concept refers to the tumor neoantigen load as an important regulator of anti-tumor immune response and a marker for response to treatment [150,151]. Along these lines, a recent study showed that BRCA 1 and 2 mutated ovarian tumors are characterized by increased neoantigen load and that this correlates with increased number of TILs, increased expression of PD1 and PDL1, and is linked to improved clinical outcome [152]. These data support exploring PD1 blockade in OC and continued investigation of the complex immune milieu associated with ovarian tumors. Therefore, identifying rational combinations to enhance the activity of PD1 blocking antibodies in OC and further analysis of the immune tumor milieu to identify predictive markers is necessary. Our group is exploring the combination of the PD1 inhibitor pembrolizumab and the DNA hypomethylating agent guadecitabine in women with recurrent platinum-resistant ovarian cancer (NCT02901899), testing the hypothesis that epigenomic priming will enhance the activity of immune checkpoint inhibitors.

## 10. Targeting Tumor Associated Macrophages (TAMs) and Myeloid-Derived Suppressor Cells (MDSCs)

Myeloid cells are frequently observed in the stroma of growing tumors [153]. The role of myeloid suppressor cells has been recognized first in late 1970s. In 2007, the term myeloid-derived suppressor cells (MDSCs) was coined for “bone marrow-derived cells of myeloid lineage comprising myeloid precursors and immature macrophages, granulocytes, and DCs, characterized by their high potential to suppress T cells” [154]. Immature myeloid suppressor cells were shown even earlier to accumulate in a variety of immune-related diseases, including cancer [155,156]. MDSC subsets were found to be responsible for immune suppression in 10 pre-clinical models of tumorigenesis [157]. In OC, macrophages are mainly found in ascites or infiltrate of the omentum. TAMs in the omentum were shown to harbor predominantly the M2 phenotype and to facilitate tumor progression [158,159]. Peritoneal TAMs support this process by secreting cytokines such as IL-6 and IL-8 [160]. In the ascites, M2 macrophage-like TAMs were found in the center of spheroids, where they participated in mechanisms supporting tumor cell proliferation and migration during OC metastasis [161]. The main signaling pathway involved in TAMs cross-talk to floating spheroid cancer cells was EGF–EGFR. TAMs promoted cancer cell invasiveness by activating the NF-κB and JNK signaling pathways [162]. Reversely, peritoneal macrophages were shown to adopt the M2 phenotype under the influence of OC cells expressing homeobox gene HOXA9 [163]. PD-L1 was primarily expressed by CD68^+^ TAMs rather than tumor cells in HGSOC, and often colocalized with both cytotoxic T cells as well as T regulatory cells and was a positive prognostic marker [146]. 

The contribution of MDSCs defined as harboring Lin^−^CD45^+^CD33^+^ markers combination was studied in a cohort of patients with HGSOC [164]. MDSCs comprised 37% of non-neoplastic cells in the TME and were responsible for inhibiting T-cell immunity, by blocking both T cell proliferation and effector function. Increased tumor MDSCs inversely correlated with CD8^+^ TILs and overall survival in advanced OC [165]. Interestingly, the corresponding Lin^−^CD45^+^CD33^+^ fraction in patients’ blood did not have the same properties. MDSCs were shown to support metastasis and a cancer stem cell phenotype. Mechanistically, it was shown that tumor-resident MDSCs enhance stemness via microRNA101, which targets co-repressor gene C-terminal binding protein-2 (CtBP2) 3’-UTR region and interferes with its binding at NANOG, OCT4/3, and SOX2 promoters in primary OC cells [164]. Primary ovarian tumors expressing high levels of Snail were shown to recruit increased number of CD33^+^ MDSCs through secretion of the CXCR2 ligands CXCL1/2 [166,167]. Therefore, blocking CXCR2 would represent a therapeutic approach for Snail-high OC tumors.

Targeting immature myeloid cells and their cross-talk with other immune cells and cancer cells is a potential strategy of combating tumor progression. Several classes of therapeutics targeting MDSCs or TAMs have been described and were recently reviewed [167]. They include agents which promote MDSCs apoptosis, antibodies that induce MDSCs and/or TAMs depletion, compounds that induce immature myeloid cells differentiation (such as retinoic acid, vitamin D3 or HDACi), inhibitors of immune suppression function (sildenafil, triterpenoids, inhibitors of COX-2, inducible nitric oxide), compounds which block recruitment (by targeting chemokines and chemokine receptors) or MDSCs proliferation, and lastly TAM reprogramming factors. Given that TAMs and MDSCs mediate resistance to immunotherapy targeting, this immune suppressive cell population could increase the success rate of checkpoint blockade inhibitors [168].

Several strategies have been tested in preclinical models, but progress towards clinical is still ongoing. For example, almetuzumab, which targets CD52 expressed by vascular leukocytes and Tie2^+^ monocytes, was shown to have anti-myeloid and anti-angiogenic properties in OC models [169]. Anti-CD52 therapy decreased tumor growth in an OC murine model. Additionally, ovarian TAMs express high levels of folate receptor-2, which can be targeted by using methotrexate loaded G5-dendrimers (G5-MTX) [170]. Noteworthy, these G5-MTX nanoparticles were shown to overcome resistance to anti-VEGF-A therapy in OC preclinical models. Epigenetic modulators have also been shown to alter the myeloid population, triggering anti-tumor immune responses. For example, the bromodomain inhibitor JQ1 significantly reduced PD-L1 expression on TAMs and dendritic cells, induced increased T cell cytotoxic activity and suppressed OC tumor growth in preclinical models [171]. A combination of histone deacetylase inhibitors (HDACi) and DNA methyltransferase inhibitor (DNMTi) was shown to reduce TAMs and increase T and NK cell activation, delaying tumor progression in preclinical models [172]. The combination of DNMTi/HDACi also synergized with the immune checkpoint inhibitors. Clinical trials testing HDACi and DNMTi with anti-PD1 therapy in patients with recurrent OC are ongoing. Lastly, catumaxomab is a humanized antibody that targets three different cell types: tumor cells (via epithelial cell adhesion molecule (EpCAM) binding); T-cells (via CD3 binding); and accessory cells (macrophages, dendritic cells, and natural killer cells) via type I, IIa, and III Fcγ receptors (FcγR). Subsequently, catumaxomab induces several effects, including T-cell-mediated tumor lysis, antibody-dependent cell-mediated cytotoxicity, and phagocytosis via activation of NK cells and TAMs. Catumaxomab is administered intra-peritoneally and was shown to be clinically active in patients with malignant ascites, leading to its approval in Europe for the treatment of EpCAM^+^ tumors associated with ascites, including HGSOC [173].

## 11. Conclusions

New targets at the interface between HGSOC cells and the TME have been characterized. Targeted treatments, alone or in combination with chemotherapy, are emerging and, in some situations, are already impacting clinical outcomes in women with HGSOC. Anti-angiogenic therapy in combination with chemotherapy has significantly improved the survival of women with advanced OC and has become part of the standard approach. In contrast, CAFs-directed strategies or therapeutics targeting cell adhesion to the matrix remain less impressive. Future development of combination and sequencing strategies based on a refined understanding of tumor biology and cross-talking pathways is critically needed. While immune interventions are still being optimized, early results suggest that combination strategies are needed to overcome the immune tolerant milieu of HGSOC. This could be due to silencing of tumor antigen and low tumor mutational burden, which render the ovarian tumors to be “cold”, or to an infiltration of immunosuppressive cells. Therefore, current approaches investigate dual immune targeting or combinations with interventions that de-repress tumor antigens through epigenetic reprogramming or which increase the tumor mutational burden by inducing DNA damage. It is clear that in order to improve clinical outcomes in this fatal malignancy, interventions affecting both cancer cells and the stroma need to be implemented. Thus, we anticipate that clinical trials will continue to explore rationally designed combinations and/or sequences of therapies targeting vulnerabilities of both tumor cells and the TME. 

## Figures and Tables

**Table 1:** Pivotal trials demonstrating Bevacizumab (Bev) clinical activity in OC.

Study	Course of Treatment	Target	TME Component	Patient Population	Phase Trial Size	Trial Endpoint	Clinical Outcome
ICON7	Chemo ± Bevac	VEGF-A	Endothelium	High risk ovarian cancer, stage IIIC or IV	Phase III N = 1528	PFS	At 42 months 22.4 vs. 24.1 months *p* = 0.04
GOG218	Chemo vs. Chemo + Bevac initiation vs. Chemo + Bevac Throughout	VEGF-A	Endothelium	New Diagnosed Stage III or IV OC	Phase III N = 1873	PFS, OS	Median PFS; 10.3 vs. 11.2 vs. 14.1 months; OS; *ns*
AURELIA	Chemo ± Bevac	VEGF-A	Endothelium	Recurrent OC PL-R	Phase III N = 361	PFS, OS	Median PFS; 3.4 vs. 6.7 months. OS; 13.3 vs. 16.6 months
OCEANS	Chemo ± Bevac	VEGF-A	Endothelium	Recurrent OC PL-S	Phase III N = 484	PFS	Median PFS 8.4 vs. 12.4 months
GOG213	Chemo ± Bevac	VEGF-A	Endothelium	Recurrent OC PL-S	Phase III N = 674	ORR	Median overall survival 37.3 vs. 42.2 months
